# New Insights and Novel Therapeutic Potentials for Macrophages in Myocardial Infarction

**DOI:** 10.1007/s10753-021-01467-2

**Published:** 2021-04-18

**Authors:** Zenglei Zhang, Junnan Tang, Xiaolin Cui, Bo Qin, Jianchao Zhang, Li Zhang, Hui Zhang, Gangqiong Liu, Wei Wang, Jinying Zhang

**Affiliations:** 1 Department of Cardiology, First Affiliated Hospital of Zhengzhou University, NO. 1 Eastern Jianshe Road, Zhengzhou, 450052 Henan China; 2 Key Laboratory of Cardiac Injury and Repair of Henan Province, Zhengzhou, Henan China; 3 Christchurch Regenerative Medicine and Tissue Engineering (CReaTE) group, Department of Orthopaedic Surgery & Musculoskeletal Medicine, University of Otago, Christchurch, 8011 New Zealand; 4 Translational Medical Center, Zhengzhou University, Zhengzhou, Henan China; 5 Henan Medical Association, Zhengzhou, Henan China

**Keywords:** macrophages, myocardial infarction, therapeutic strategies, cardiac repair, polarization

## Abstract

Cardiovascular disease (CVD) has long been the leading cause of death worldwide, and myocardial infarction (MI) accounts for the greatest proportion of CVD. Recent research has revealed that inflammation plays a major role in the pathogenesis of CVD and other manifestations of atherosclerosis. Overwhelming evidence supports the view that macrophages, as the basic cell component of the innate immune system, play a pivotal role in atherosclerosis initiation and progression. Limited but indispensable resident macrophages have been detected in the healthy heart; however, the number of cardiac macrophages significantly increases during cardiac injury. In the early period of initial cardiac damage (e.g., MI), numerous classically activated macrophages (M1) originating from the bone marrow and spleen are rapidly recruited to damaged sites, where they are responsible for cardiac remodeling. After the inflammatory stage, the macrophages shift toward an alternatively activated phenotype (M2) that promotes cardiac repair. In addition, extensive studies have shown the therapeutic potential of macrophages as targets, especially for emerging nanoparticle-mediated drug delivery systems. In the present review, we focused on the role of macrophages in the development and progression of MI, factors regulating macrophage activation and function, and the therapeutic potential of macrophages in MI.

## INTRODUCTION

Despite advances in prevention, diagnosis, and treatment, cardiovascular disease (CVD) remains the primary cause of death worldwide, and myocardial infarction (MI) makes the greatest contribution to CVD [1]. Furthermore, the World Health Organization has predicted that annual deaths from CVD will increase from 18.1 million in 2010 to 24.2 million in 2030 globally [2]. CVDs, including hypertension, atherosclerosis, ischemic heart disease like MI, and ischemic stroke [3], result in significant death and disability [4]. Considering the poor prognosis associated with CVD, new therapeutic strategies are needed to facilitate cardiac repair following MI.

Previous studies have demonstrated that vertebrate zebrafish is capable of complete cardiac regeneration [5–7], while some regenerative capacity of mammals can be maintained for just a few days [8, 9]. Macrophages are basic cell components of the innate immune system that infiltrate into injured myocardium during neonatal heart regeneration [8]. Accumulating evidence has revealed that inflammation plays a major role in the pathogenesis of coronary artery disease and atherosclerosis [10–12] and is necessary for correct and timely repair [13, 14]. After MI, circulating blood monocytes rapidly infiltrate into the infarcted area and differentiate into the appropriate macrophages [15]. Based on surface markers and functions, macrophages are divided into two major subtypes: classically activated macrophages (M1), which are related to inflammatory response, and alternatively activated macrophages (M2), which are associated with regeneration and injury repair. In the inflammatory phase of MI, M1 macrophages activated by tumor necrosis factor-alpha (TNF-*α*), interferon-gamma (IFN-*γ*), and lipopolysaccharide (LPS) are the leading subtypes that initially respond to the removal of dead cells and cellular and extracellular matrix (ECM) debris [16, 17]. In the proliferative phase of MI, M2 macrophages gradually predominate to facilitate the repair and regeneration of damaged cardiac tissues [18, 19]. Therefore, the correct and timely regulation of macrophage polarization is a promising therapeutic target for the treatment of MI. In addition, stem cell transplantation and nanoparticle-mediated drug delivery systems have made extensive breakthroughs. Together, macrophages modulated by all kinds of therapeutic strategies, particularly the nanoparticle-mediated drug delivery system, have become the promising therapeutic target in the field of cardiac repair.

**Fig. 1:**
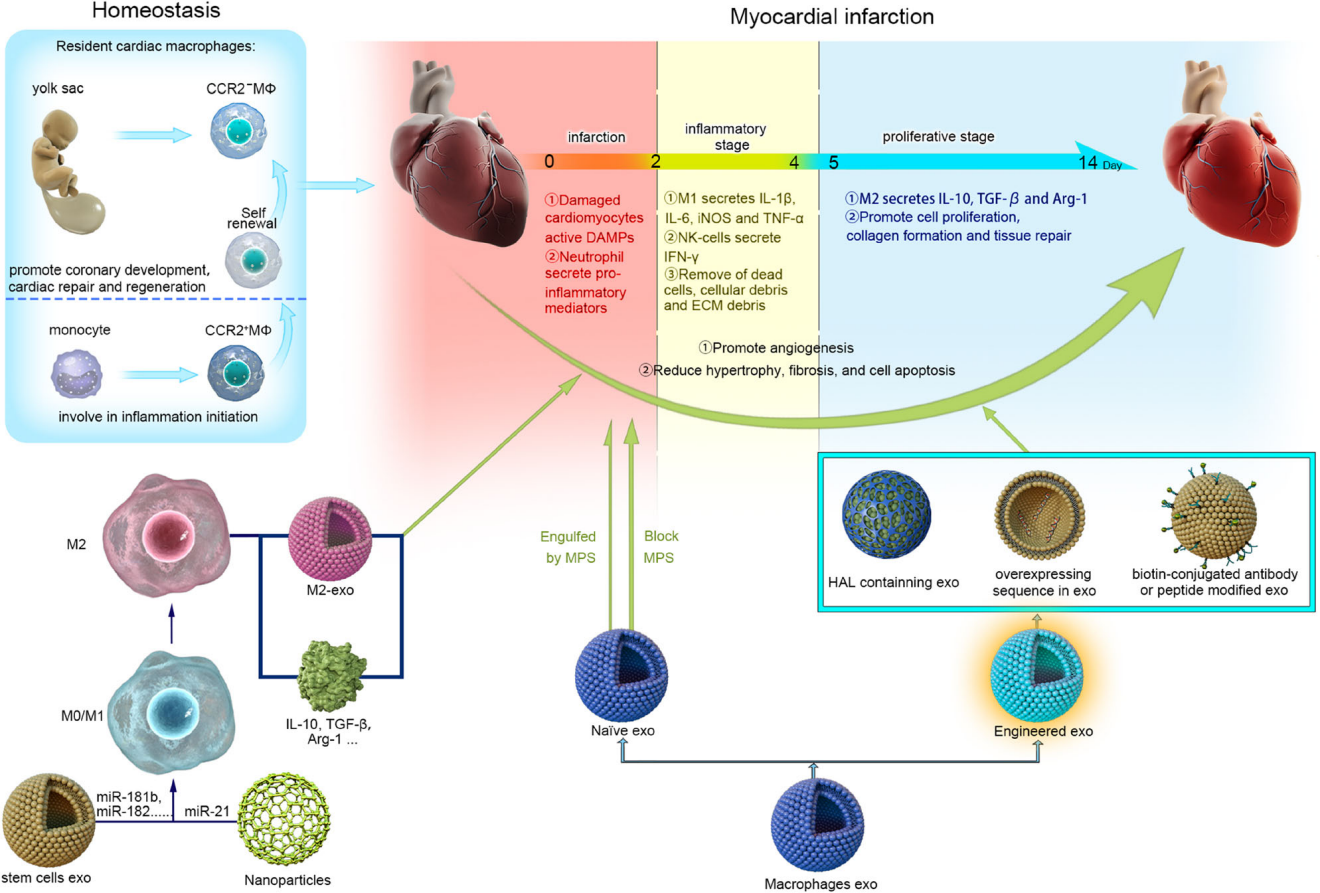
Macrophages function in homeostasis and in/post-myocardial infarction. The three overlapping stages are involved in the repair response after obstruction of the blood flow: infarction, inflammatory, and proliferative. In the infarction phase, damaged cardiomyocytes, active DAMPs, and neutrophils are recruited to the infarcted site and release many inflammatory mediators, which are indispensable for the subsequent inflammatory stage. In the inflammatory stage, proinflammatory subset M1 macrophages and NK cells secrete inflammatory cytokines, such as IL-1*β*, IL-6, iNOS, TNF-*α*, and IFN-*γ*, which promote clearance of dead cells and cellular and ECM debris. Last, the proliferative stage involves cell proliferation, collagen formation, and tissue repair that mainly contribute to anti-inflammatory subset M2 macrophages that secrete IL-10, TGF-*β*, and Arg-1. Modulation (i.e., self-assembly/engineered extracellular vesicles including exosomes[exo], nanoparticles, stem cells) of macrophages may repair damaged myocardium by promoting angiogenesis and reducing hypertrophy, fibrosis, and cell apoptosis.

In the present review, we discuss recent findings on the association of macrophages with the development of post-MI. More specifically, we focus on the phenotypes and functions of macrophages in a steady state and during MI, as well as possible mechanisms underlying macrophage polarization in the heart. Importantly, we discuss potential therapeutic strategies to improve injury control and functional recovery by modulating macrophage polarization, which involves self-assembly/engineered extracellular vesicles (EVs), nanomedicine, and stem cells (Fig. 1).

## ONTOGENY OF CARDIAC MACROPHAGES

Macrophages are the first immune cells that develop during the development of an organism. They play a crucial role in immunity (homeostasis and inflammation) and also regulate organ development and function [20]. Macrophages primarily originate from circulating blood monocytes [21]. Monocytes pertain to the population of mononuclear leukocytes derived from hematopoietic stem cells in fetal liver, adult bone marrow, and splenic reservoir under the stimulation of some cytokines, such as M-CSF, GM-CSF, interleukin-1*β* (IL-1*β*), and IL-3 [22, 23], and are released in the bloodstream. Monocytes can be divided into two classifications after being mobilized into the peripheral circulation [24]: (1) Ly6C^l^° CCR2^−^ CX3CR1^hi^ patrolling monocytes (CD14^l^°CD16^+^ in humans), which are responsible for surveying the vascular lumen and for clearing dead cells and cellular and ECM debris, and (2) Ly6C^hi^ CCR2^+^ CX3CR1^l^° inflammatory monocytes (CD14^hi^CD16^−^ in humans), which produce proinflammatory cytokines, such as IL-1, IL-10, and TNF-*α*. Over the past 40 years, it has been accepted that all forms of macrophages, including resident macrophages, originate from monocytes; however, this has been challenged in the past few years [25]. Recent research has shown that tissue-resident macrophages represent a different population of cells that are derived from diverse lineages [21, 26, 27]. Studies using mouse models have revealed that many tissue-resident macrophages in the kidney, lung, skin, brain, liver, and heart originate from an embryonic lineage and are maintained throughout life free of monocyte recruitment [28–32]. In recent years, advances in gene fate-mapping techniques have revealed the two distinct populations of tissue-resident macrophages originating from the prenatal yolk sac and fetal liver [28, 29]. It is now clear that the majority of cardiac resident macrophages originate from the yolk sac [30, 31, 33], and these cardiac resident macrophages are divided into two populations that coexist at homeostasis: MHC-II^l^°CCR2^−^ and MHC-II^hi^CCR2^−^ cells. The others are known as circulating monocyte-derived MHC-II^hi^CCR2^+^ cardiac resident macrophages that are responsible for inflammation initiation (Table 1).

**Table 1:** Origins and Function of Cardiac Resident Macrophages

Surface markers	Origin	Function	References
MHC-II^lo^CCR2^-^	Yolk sac	Phagocytic ability of dying cardiomyocytes	30-31,33
MHC-II^hi^CCR2^-^	Yolk sac	Efficiently processed and presented antigen to T cells	30-31,33
MHC-II^hi^CCR2^+^	Circulating monocytes	Inflammation initiation and efficiently processed and presented antigen to T cells	25,33

## MACROPHAGES: INFLAMMATION, ACTIVATION, AND FUNCTION

**Fig. 2:**
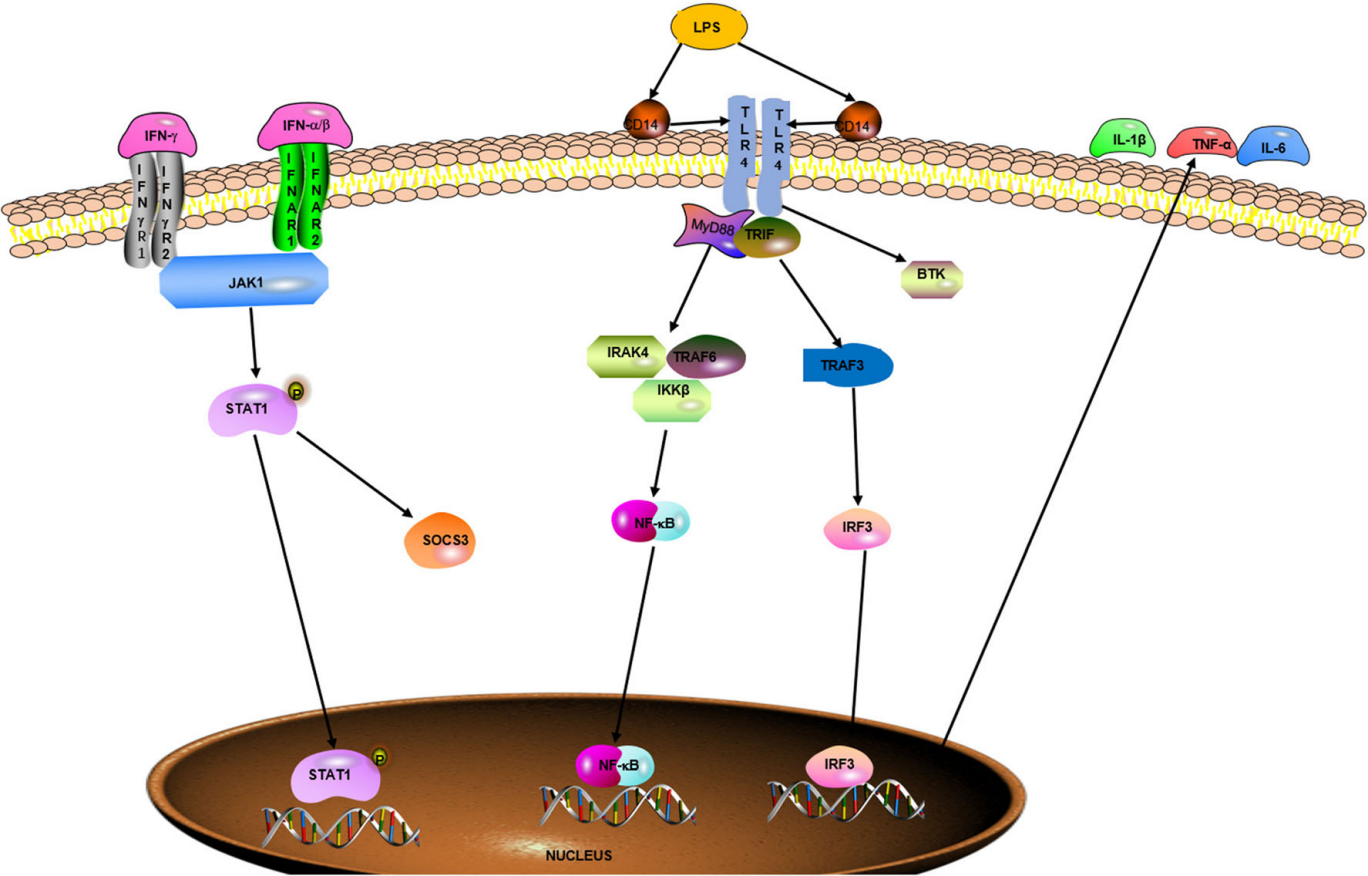
Signaling pathways regulating M1 macrophage polarization. Naïve macrophage is induced into M1 macrophage by LPS, IFN-*γ*, and IFN-*α*/*β* through specific receptors, such as TLR4, IFN*γ*R, and IFNAR. And the related signaling pathways such as STAT1, NF-κB, and IRF3 have an important role in the process, which results in the secretion of proinflammatory cytokines, such as IL-1*β*, TNF-*α*, and IL-6.

**Fig. 3:**
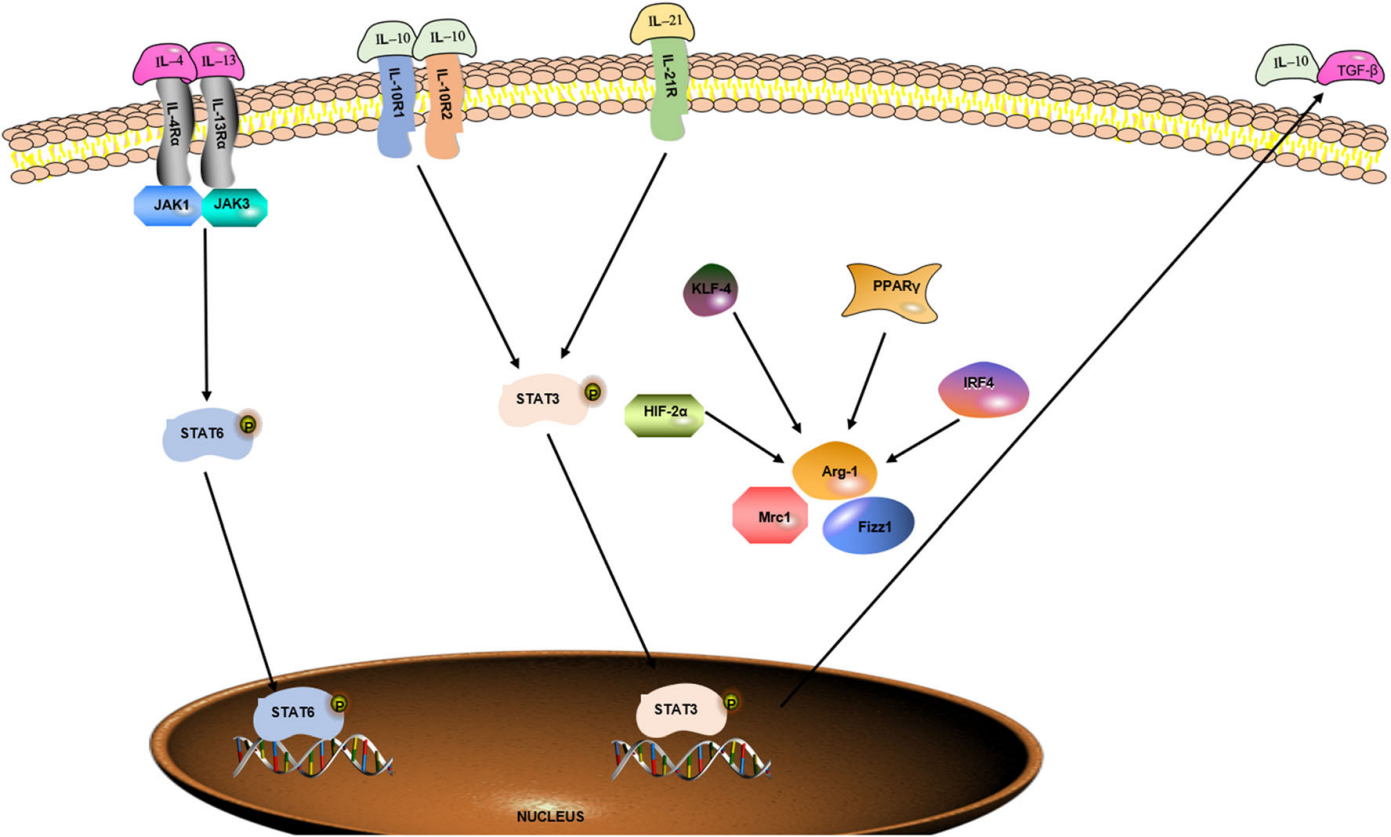
Signaling pathways regulating M2 macrophage polarization. Naïve macrophage is induced into M2 macrophage by IL-4, IL-13, IL-10, and IL-21 through interacting with specific receptors, such as IL-4R*α*, IL-13R*α*, IL-10R, and il-21R. And the related signaling pathways such as STAT6, STAT3, HIF-2*α*, KLF-4, PPAR*γ*, and IRF4 are activated in the process, which promotes the secretion of anti-inflammatory cytokines, such as TGF-*β* and IL-10.

Macrophages, as the basic cell component of the innate immune system, are involved in all stages of atherosclerosis. Macrophages exhibit extensive functional plasticity that is dependent on activation (*in vitro*) or microenvironmental milieu (*in vivo*). Macrophages not only possess the essential functions of phagocytic killing of pathogens and antigen presentation activating an adaptive immune response but also maintain tissue homeostasis by eliminating dead cells, cellular debris, and ECM debris and by promoting adaptive remodeling of the ECM [34]. Macrophages acquire a proinflammatory or an anti-inflammatory subtype under proper environmental stimuli [34]. Therefore, macrophages have been divided into M1 and M2 [35, 36] based on the type of *in vitro* stimulation, surface molecule expression pattern, secretory profile, and function. Although this distinction has deficiencies in adequately including the entire macrophage biological complexity, it provides a common scheme to classify macrophage function. In addition, the switch of macrophage polarization is tightly regulated by signaling networks at the transcriptional and translational levels (Figs. 2 and 3).

M1 macrophages are characterized by increased microbicidal or tumoricidal capacity and secrete high amounts of proinflammatory cytokines and mediators. Classically activated M1 macrophages are triggered by IFN-*γ* and LPS and are characterized by upregulated biomarkers, such as IL-1*β*, IL-6, IL-12, and IL-23; inducible nitric oxide synthase (iNOS); TNF-*α*; chemokine (C-C motif) ligand 2 (CCL2), CCL15, and CCL20; C-X-C motif chemokine 9 (CXCL9), CXCL10, and CXCL11; and CD80 and CD86 [37–39]. M1 macrophages detect and recognize damage-associated molecular patterns (DAMPs) present in the debris of necrotic cells and pathogen-associated molecular patterns (PAMPs) such as LPS and chitin through toll-like receptors (TLRs) located on the surface of macrophages to promote inflammation response. During an innate immune response, TLR agonists engage the MyD88-dependent pathway, including IRAK4, TRAF6, and IKK*β*, ultimately leading to the activation of NF-κB to induce M1 polarization [40]. In addition, a TLR ligand can induce the transcription of TNF through an MyD88-dependent manner, further cooperating with IFN-*γ* in an autocrine manner to activate these macrophages [34]. IFNs mediate the activation of IRF/STAT signaling pathways *via* the JAK/STAT signaling pathway, favoring M1 polarization [41, 42]. IFN-*γ*, which is produced by natural killer (NK) cells responding to stress and infections, can promote macrophages to produce proinflammatory cytokineswhich in turn increase the killing ability of NK cells [43]. Typically, research has shown that mice and humans who lack IFN-*γ* expression are more susceptible to protozoal and certain kinds of bacterial or viral infections [44]. Furthermore, activated M1 macrophages by LPS *via* an exogenous TLR ligand can clear the parasite completely [34]. Finally, M1-secreted proinflammatory cytokines play an important role in host defense but also can cause extensive damage to the host.

M2 macrophages are characterized as having wound healing and proliferative properties. M2 macrophages, which are activated by the stimulation of IL-4, IL-13, or IL-21, are involved in wound repair, homeostasis, and tumor metastasis and tumor promotion. They also secrete anti-inflammatory cytokines [45–47]. M2 macrophages are characterized by decreased expression levels of biomarkers, such as TGF-*β*, CD163, CD206, chil3 chitinase-like 3 (known as Ym-1), resistin-like-*α* (known as Fizz1), arginase 1 (Arg-1), and IL-10 to promote cell proliferation, collagen formation, and tissue repair [47]. Aberrant activation of M2 macrophages is associated with tissue fibrosis. Accumulating evidence has indicated that macrophages lacking expression of IL-4 receptor (IL-4R) are incapable of promoting wound healing. In terms of mechanism, M2 macrophages can be triggered by IL-4/L-13 and IL-10/IL-21, depending on the activation of the IRF/STAT signaling pathway *via* STAT6 and STAT3 [48–50]. IRF4, PPAR*γ*, Krüppel-like factor 4 (KLF-4), and HIF-2*α* also mediate the induction of the M2 phenotype [40]. M2 macrophages are further divided into different phenotypes denoted by the stimulus and effector function. IL-4 and IL-13 can induce M2a polarization, whereas Fc-*γ* receptors and TLR stimulation can trigger M2b macrophages, and GC, IL-10, or TGF-*β* ligands are responsible for M2c activation [37]. In function, both M2a and M2c can enhance the adaptive immune response, whereas M2b plays a key role in suppressing and regulating inflammation and immunity [51].

## MACROPHAGES IN CARDIAC HOMEOSTASIS

Cardiac resident macrophages, which account for 6% to 8% of the noncardiomyocyte population according to the data from healthy adult mouse heart, are indispensable for maintaining the cardiac homeostasis and neonatal heart regeneration [52]. The long-held perspective is that the majority of cardiac resident macrophages originate from peripheral blood monocytes and present an M2 polarization profile [53]. Recently, numerous researchers have demonstrated that cardiac resident macrophages consist of a heterogeneous population that includes resident macrophages derived from the yolk sac: MHC-II^l^°CCR2^−^ and MHC-II^hi^CCR2^−^ cells, macrophages derived from fetal monocytes and macrophages derived from adult monocytes [15, 33, 54, 55]. The populations seed the heart at specific developmental stages so that they can maintain cardiac homeostasis.

As noted, yolk sac–derived CCR2^−^ macrophages play a major role in the coronary development and cardiac repair. Deletion of CCR2^−^ macrophages in the embryonic period could cause abnormal remodeling, diminished LV systolic function, larger LV chamber dimensions, and increased akinetic myocardium [56]. Circulating monocyte-derived CCR2^+^ cardiac resident macrophages are abundant in proinflammatory genes involved in inflammation initiation and exhibit an M1 polarization profile to maintain cardiac homeostasis. Interestingly, more substantial contributions of circulating monocytes have been observed in the aging heart, indicating that circulating blood monocytes may differentiate into CCR2^−^ macrophages [57, 58], which seemingly coincide with the decreased self-renewal ability of yolk sac–derived resident CCR2^−^ macrophages with age, as demonstrated by Molawi et al. [58].

## MACROPHAGES IN THE PROCESS OF ATHEROSCLEROSIS AND THROMBUS FORMATION

Atherosclerosis is considered to be a chronic inflammatory disease. Perturbation of lipid metabolism and local inflammation are the two major causes in the pathogenesis of atherosclerosis, including cells such as platelets, which play an initiating role in the development of atherosclerosis and the atherosclerotic plaque [59], monocytes and endothelial cells, connective-tissue elements, lipids, and debris. First, low-density lipoproteins (LDLs) accumulate in the intima, activating endothelial cells to initiate atherosclerosis formation [60]. This accumulation of lipoproteins is located in the subendothelial space and is modified by reactive oxygen species and enzymatic cleavage to be involved in the inflammatory process [61]. LDL, especially oxidized LDL (ox-LDL), and activated platelets are responsible for recruiting circulating monocytes into the endothelial space where they are further differentiated into macrophages that engulf ox-LDL and LDL to form foam cells, which finally produces proinflammatory cytokines (e.g., TNF-*α*, IL-1, and IL-6) and exacerbate local inflammation [62]. In addition, Lindemann et al. reported that progenitor cells adhere to lipid-laden platelets and turn into macrophages that internalize the lipid-rich platelets and develop into foam cells [63]. Next, macrophages, mast cells, and T cells infiltrate the atherosclerotic plaques, exhibit signs of activation, and secrete inflammatory cytokines responding to proinflammatory cytokines [10]. The apoptosis of foam cells in the lipid core is a key reason for atherosclerosis progression. Next, these apoptotic foam cells are removed primarily by macrophages, a process known as efferocytosis. Further inflammation, necrosis, and thrombosis originating from the gradual accumulation of apoptotic debris in the lipid core have occurred when the balance in which efferocytosis internalizes all dead cells is disturbed [64].

## THE ROLE OF MACROPHAGES DURING AND POST-MYOCARDIAL INFARCTION

MI is defined as pathologically myocardial cell death resulting from prolonged myocardial ischemia. MI is the leading cause of mortality and disability worldwide [1] and results in a major socioeconomic burden. Such events result from the imbalance between myocardial oxygen supply and demand. Although multiple other mechanisms have been reported to contribute to MI, coronary thrombosis promoted by rupture of the atheroma plaque accounts for most of the cases of MI [65]. Cardiomyocyte necrosis resulting from coronary artery ischemia triggers both a systemic inflammatory response and a local reaction to recruit circulating monocytes into the infarcted site. The post-MI repair response includes three sequential stages: inflammation, tissue replacement, and healing or maturation. In the early inflammatory stage, infiltrated neutrophils and recruited circulating monocytes and cardiac resident macrophages contribute to clear the dead cells and matrix debris. Next, these inflammatory responses are gradually replaced by proliferative monocytes and macrophages, which results in angiogenesis and myofibroblast differentiation. Finally, in the healing phase, fibroblasts, immune cells, and microvasculature form a mature scar [66]. In the following phases, we analyze the role of macrophages in the inflammatory and healing stages after MI.

### M1 Macrophages in Myocardial Infarction

MI can lead to necrosis of cardiac myocytes within a few minutes. To maintain tissue integrity and function, inflammatory cells, including neutrophils and macrophages, are activated. After MI, neutrophils are the first immune cells to occur in the infarcted area in large numbers and are responsible for clearing cellular debris and further recruiting leucocytes such as Ly-6C^hi^ monocytes and macrophages [67, 68]. Monocytes and macrophages are the two major cell populations infiltrating the damaged site. Depletion of monocytes and macrophages may result in a thromboembolic event [69]. The two sequential monocyte and macrophage phases demonstrate a significant difference in healing after MI: first, bone marrow– and spleen-derived Ly-6C^hi^ monocytes are recruited to the infarcted site *via* MCP-1. Its receptor, CCR2 [70], and corresponding M1 macrophages predominate in the infarcted region during days 2 to 5 post-MI. During the inflammatory phase of MI, significant proinflammatory mediators, such as TNF-*α*, IL-1*β*, and proteases, originating from activated Ly-6C^hi^/M1, are released in the damaged site, which contributes to the clearance of dead cells and debris in the infarcted region through the activated M1 macrophages. The process of phagocytosis is indispensable for proper initiation of the wound repair after MI [71]. However, prolonged inflammatory response to such compounds can result in extensive damage to infarcted myocardium.

### M2 Macrophages in Myocardial Infarction

MI results in necrosis of cardiac myocytes within a few minutes, contributing to chamber dilatation, contractile dysfunction, and eventually heart failure. The regenerative capacity of mammals persists for only a short period [8, 9]. Therefore, the correct and timely repair after MI is necessary to maintain the constructive and functional integrity of the heart. Macrophages can promote the infarcted repair as regulators and effectors. In addition, different effects on fibrosis and scarring versus regeneration as a result of the depletion of macrophages at different stages post-injury in a model of liver fibrosis have been demonstrated [72].

From days 4 to 14 post-MI, Ly-6C^hi^ monocytes and M1 macrophages are replaced by the Ly-6C^low^ monocytes and M2 macrophages. The proliferative phase after MI origin from the macrophages shifts from inflammatory (M1) to reparative phenotypes (M2). M2 macrophages gradually dominate in the infarcted sites. Then, M2 macrophages establish an anti-inflammatory environment by downregulating inflammatory cytokines and upregulating anti-inflammatory cytokines, such as IL-10, VEGF, and TGF-*β* [73]. TGF-*β* and IL-10 can trigger myofibroblasts to produce collagen, and VEGF promotes cell proliferation and blood vessel development. The lack of the Trib1 gene in the myocardial-infarcted mouse model presented selective deletion of M2 macrophages, which resulted from an impaired ability to form M2 macrophages in the spleen, liver, and adipose tissue. Compared with a control group, diminished reparative function after MI, similar to frequent cardiac rupture, was observed. In addition, IL-4 has been demonstrated to improve the post-MI prognosis of wild-type mice with an increased number of M2 macrophages. Together, M2 macrophages play a vital role in facilitating myocardial wound healing in adult murine heart [74]. Moreover, in the GR^LysMCre^ mice, Galuppo et al. reported that the glucocorticoid receptor in macrophages critically determines post-MI repair by regulating myofibroblast differentiation in the infarct microenvironment during the early phase of wound healing [75].

## THE ROLE OF MACROPHAGES IN MYOCARDIAL ISCHEMIA/REPERFUSION INJURY

As noted, the most timely and effective treatment involves amelioration of myocardial ischemia and restriction of the size of MI. Ischemia/reperfusion (I/R) such as percutaneous coronary intervention and intravenous thrombolysis has so far been the principal or only strategy for MI treatment, thereby promptly restoring blood supply [76]. However, deteriorated ischemic damages and further swelling of the infarct size will be accompanied with sudden reperfusion, which results in secondary cascade damages, known as myocardial I/R injury [77]. I/R injury may trigger all kinds of pathological changes, including local acute inflammatory reactions, metabolic disorders, and cell apoptosis or necrosis, even resulting in cardiac dysfunction. Macrophages, a major type of inflammatory cells, have a crucial effect on myocardial ischemic injury with reperfusion [78] and have multiple roles because of their specific phenotypes and the stage of disease.

### M1 Macrophages in Myocardial Ischemia/Reperfusion Injury

Though M1 macrophages are believed to damage the heart in the early period of reperfusion by releasing reactive oxygen species, inflammatory mediators, and proteases [79, 80], some researchers have found that the process of phagocytosis performed by the M1 macrophages is essential for further repair. Fan et al. reported that M1 macrophages polarized by Dectin-1 expressed largely on cardiac macrophages aggravate myocardial I/R injury [81]. In contrast, a previous study has reported that soluble receptor for advanced glycation end products can improve heart function in mice after I/R by promoting infiltration and differentiation of macrophages into M1 and IFN-*γ* production [82]. M1 macrophages may involve cardioprotection primarily in the period of ischemia while damaging the heart in the following stages by releasing inflammatory cytokines and recruiting the inflammatory cells. In clinical practice, therapeutic strategies are applied mainly to recover blood flow in a timely manner and ameliorate myocardial I/R injury. Thus, methods limiting M1 while promoting M2 polarization of macrophages in myocardial I/R injury have been researched extensively and represent a unique therapeutic strategy to suppress inflammatory responses and ameliorate myocardial I/R injury.

### M2 Macrophages in Myocardial Ischemia/Reperfusion Injury

M2 macrophages, polarized by Th2 cytokines and characterized by the production of high levels of anti-inflammatory cytokines and pro-fibrogenic factors, exhibit anti-inflammatory and tissue repair properties. Accumulated evidence has demonstrated that M2 macrophages play an important role in alleviating myocardial I/R injury. A previous study reported that alternatively activated M2 macrophages by Chemerin15 protect against myocardial I/R injury in mice by significantly suppressing proinflammatory cytokines and markedly increasing the level of anti-inflammatory cytokine IL-10 [83]. In recent years, M2b macrophages, as one subtype of M2 macrophages and regulatory cells, have drawn considerable attention for the treatment of myocardial I/R injury. *In vivo* experiments have shown that transplantation of M2b macrophages into the myocardium that had been subjected to I/R injury improved cardiac function and reduced the cardiac fibrosis and myocardial remodeling caused by I/R injury [84]. Yue et al. reported that M2b macrophages modulate inflammatory immune responses without participating in wound healing and enhance protective effects on myocardial remodeling after myocardial I/R injury [85].

## TARGETING THE THERAPEUTIC ROLE OF MACROPHAGES POST-MI

### Exogenous Cardioprotection by Modulation of Macrophage Polarization

It has been reported that stem cells and stromal cells could be utilized for the treatment of myocardial injury by modulating macrophage polarization. Cardiosphere-derived cells (CDCs), essentially cardiac stromal cells (CSCs), represent a promising stem cell source for repairing damaged heart tissue [86–88]. These cells can regulate macrophage activation, leading to the promotion of a phenotypic switch from M1 to M2 [89, 90]. In a CDC-treated MI model, functional and structural benefits, such as decreased infarcted area, improved cardiac function, and enhanced angiogenesis after MI, have been observed by modulating M1/M2 macrophage polarization and neutrophil recruitment [89–91]. Human embryonic stem cell–derived cardiovascular progenitor cells (hESC-CVPCs) also are known to be attractive cell sources for cardiac repair. hESC-CVPCs, which modulate cardiac macrophages toward an M2 phenotype, play an important role in ameliorating worsening heart function and reducing scar formation through a paracrine effect–activated STAT6 [92]. In addition, many studies have demonstrated that mesenchymal stem cells (MSCs) play a key role in post-MI repair by reversing cardiac dysfunction and enhancing angiogenesis, which may result from regulating the M1/M2 balance [93, 94]. MSCs have been reported to regulate a macrophage subtype toward an M2-like status *in vitro* and *in vivo* [95].

### EVs Mediate Cardioprotection in MI

EVs have been demonstrated to play a crucial role in cell-cell communication during different pathological and physiological processes [96, 97]. EVs have received increasing attention as cell-free therapeutics for regenerative medicine because of the structure of their lipid bilayer and cargos, such as miRNA, protein, and lipids [98–101] (Table 2). As noted earlier in this review, CDCs and MSCs can promote M2 polarization in cases in which the EVs may play, at least partly, a major role.

**Table 2:** Extracellular Vesicles Associated with Macrophage-Mediated Cardioprotection

Cell source	Disease model	Injection method	Contents	Mechanism	Biological effects	Reference
Mouse BM-MSCs	Mouse myocardial I/R model	Intramyocardial injection	miR-182	Polarizes MΦ into M2 subtype *via* downregulating TLR4/NF-κB and upregulating PI3K/Akt	Attenuates myocardial I/R injury	102
Human BM-MSCs	Mouse MI model	Intravenous injection	miR-101a	Polarizes MΦ into M2 subtype	Preserves cardiac function and reduces scar size	104
Human CDCs	Rat and pig myocardial I/R model	Intramyocardial injection	miR-181b	Polarizes MΦ into M2 subtype *via* inhibiting the expression of PKCδ	Reduces infarct size and alleviates cardiac inflammation	105
Rat and mouse CDCs	Rat and mouse myocardial I/R model	Intramyocardial injection	Whole content	Modulates the expression of MerTK and C1qa	Attenuates irreversible damage	107
Human CDCs	Rat myocardial I/R model	Intramyocardial injection	Y RNA fragment	Polarizes MΦ into M2 subtype	Reduces infarct size	109
M2 macrophages	Rat myocardial I/R model	Intramyocardial injection	miR-148a	Inhibits TXNIP expression	Alleviates myocardial I/R injury	112

Abbreviations: *BM*, bone marrow; *CDCs*, cardiosphere-derived cells; *I/R*, ischemia/reperfusion; *MI*, myocardial infarction; *miR*, microRNA; *MSCs*, mesenchymal stromal cells; *MΦ*, macrophage; *PKCδ*, protein kinase C *δ*

MSCs have long become a promising therapeutic strategy for ischemic heart disease, although the mechanism remains elusive. Several recent studies have implicated that MSC-exo could polarize macrophage to create an anti-inflammatory environment under myocardial I/R injury or MI [102, 103]. MSC-exo, which transfers miR-182 and miR-101a into macrophage, has been demonstrated to reduce the number of M1 macrophages, polarize macrophages into M2 phenotype, and reduce infarct size and inflammatory response under myocardial I/R injury [102, 104].

CDC or CDC-derived EVs polarize the macrophage into a special phenotype that is highly phagocytic and anti-inflammatory to display cardioprotection [105, 106]. De Couto et al. further revealed that CDC-derived EVs enhance macrophage efferocytosis and cardioprotection response *via* EV transfer of miR-26a to modulate the expression of MerTK and C1qa [107]. Moreover, miR-181b–enriched exosomes secreted from CDCs play a critical role in modulating macrophage polarization *in vitro* and confer cardioprotection *in vivo* by minimizing infarct size, decreasing the total number of CD68^+^ macrophages, and inducing macrophages to develop into a distinct subtype [105]. Another study has suggested that CDC-derived EVs can shift M1 macrophage into the pro-angiogenic subtype [108]. Furthermore, the short non-coding RNA, Y RNA fragment (the Y RNAs consist of 83–112 nucleotides known as one poorly understood class of non-coding RNA), is particularly plentiful in EVs and was first discovered in complex with ribonucleoproteins in the serum of patients with lupus. The characteristic stem-loop secondary structure and high sequence conservation between the upper and lower stem have been found in the four human Y RNAs. Highly enriched CDC-derived EVs also have a crucial cardioprotection response by promoting IL-10 gene expression and secretion in macrophages [109].

Nguyen et al. reported that atherogenic macrophage-secreted EVs can inhibit macrophage migration *via* transferring miRNAs, especially the miR-146a, which downregulates target genes IGF2BP1 and HuR in recipient cells, thereby promoting the progression of atherosclerosis [110]. Moreover, miR-155–containing exosomes derived from activated macrophages are adverse to post-MI repair. A recent study has shown that exosomes inhibit cardiac fibroblast proliferation and promote inflammation by downregulating Son of Sevenless 1 expression and decreasing suppressor of cytokine signaling 1 expression, while *in vivo* experiments revealed a lower incidence of cardiac rupture and improved cardiac function in the miR-155–deficient mice compared with controls [111]. In addition, M2 macrophages secreting miR-148a–enriched exosomes can reduce the size of the infarct and improve cardiac function following MI [112]. Furthermore, Wu et al. obtained molecularly engineered M2 macrophage exosomes, which were further electroporated with a US Food and Drug Administration (FDA)–approved hexyl 5-aminolevulinate hydrochloride (HAL). These HAL-containing M2 exosomes exhibited anti-inflammatory capabilities and ultimately alleviated atherosclerosis because of the anti-inflammatory effects of the M2 exosomes and the encapsulated HAL affect. The exosomes were involved in endogenous biosynthesis and metabolism of heme to produce carbon monoxide and bilirubin, which have known anti-inflammatory capabilities [113].

Although EVs have been demonstrated to be an attractive therapeutic approach to ischemic heart disease, low retention and short-lived therapeutic effects remain significant challenges. To reduce off-target delivery, engineered exosomes and prior blocking of endocytosis of exosomes by macrophages have been utilized to enhance the delivery efficiency of exosomes to specific cells, offering therapeutic benefit [114–116]. Thus, these have improved delivery efficiency, including platelet nanovesicles [117], monocyte mimic–modified EVs [118], EVs incorporated in alginate hydrogel [119], overexpressed targeting sequences or those modified by DMPE-PEG-streptavidin (DPS), and biotin-conjugated antibody or peptide.

### Cardioprotection Using a Nanoparticle-Mediated Drug Delivery System

To improve the safety and efficiency of therapeutic agents, specially designed nanocarriers, including liposomes, polymeric nanoparticles, and complexes, have been widely applied, particularly in the cardiovascular and oncology fields. These nanocarriers containing active substances, such as siRNAs or statins, effectively polarize macrophages into the anti-inflammatory phenotype or mediate gene expression in macrophages (Table 3). Increasing evidence suggests that these changes in macrophages promote cardiac repair in infarcted animal models.

**Table 3:** Examples of Macrophage-Based Nanoparticle-Mediated Drug Delivery Systems

Nano-carrier	Disease model	Injection method	Contents	Mechanism	Biological effects	Reference
Liposome	Rat MI model	Intravenous injection	Phosphatidylserine	Increases M2 macrophages	Promotes angiogenesis, decreases scarring, prevents ventricular dilatation and remodeling	125-126
Acid sensitive polyketal PK3 particle	Mouse MI model	Intramyocardial injection	Nox2-siRNA	Silences the Nox2 gene of cardiac macrophages	Recovers cardiac function	129
Optimized lipid nanoparticle	Mouse myocardial I/R model	Intravenous injection	CCR2-siRNA	Decrease M1 macrophages	Reduces infarct size	130

Abbreviations: *I/R*, ischemia/reperfusion; *MI*, myocardial infarction; *siRNA*, RNA-mediated silencing

#### Liposomes

Liposomes are microscopic phospholipid bubbles, which include one or more biocompatible lipid bilayers with an aqueous core inside, ranging from a few hundreds to thousands of nanometers in size [120–123]. Liposomes have been used mostly in basic and clinical medicine since the first FDA-approved liposomal drug Doxil® was used and have exhibited advantageous performance in facilitating encapsulation of a broad variety of pharmaceuticals, depending on the intrinsic amphiphilicity of their lipid bilayer shell(s) [124]. Moreover, compared with the conventional (classical) liposomes, immunoliposomes, which are target liposomes with different surface-targeted ligands, including antibodies and peptides, can target the special cells and reduce the recognition of MPS to eliminate the off-target effects. Tamar et al. demonstrated that systemic administration of phosphatidylserine (PS)-presenting liposomes significantly increases the number of CD206^+^ macrophages and the level of anti-inflammatory cytokines, such as TGF-*β* and IL-10, and downregulates the expression of proinflammatory markers at the same time, which promotes angiogenesis and halts ventricular dilatation and adverse remodeling in a rat model of acute MI [125]. Moreover, Ruvinov et al. showed a similar therapeutic effect in a rat model of acute MI by activating macrophages into an anti-inflammatory state [126].

#### Polymeric Nanoparticles and Complexes

Polymeric nanoparticles (NPs) mainly consist of biodegradable and biocompatible polymers such as natural polymers (e.g., albumin), polylactide, and poly(D,L-lactide co-glycolic acid) (PLGA) and are heterogeneous in size, often ranging from a few tens to thousands of nanometers in diameter [127]. Because of both the hydrophilic and hydrophobic character of polymeric NPs, they have been used in all kinds of pharmaceuticals. Polymeric NPs are promising candidates for drug delivery and have received public attention since the albumin-paclitaxel complex (Abraxane) was approved for IV treatment. This review describes the excellent properties of polymeric NPs: first, these prevent cargo degradation; second, they decrease phagocytosis by MPS; third, they break the absorption barrier formed by biological membranes; and last, they provide a method of sustained drug release. Even more encouraging is that PLGA-based NPs can escape the endolysosomal compartment and release the encapsulated payload in the cytoplasm following cell internalization into the cell by endocytosis [128].

Monocytes/macrophages have been known as targets that are modulated by polymeric NP– and complex-based carriers in the field of cardiac repair and have caught special attention in the past few years. Recent studies have demonstrated that systemic administration of siRNA against messenger (mRNA) loaded into NPs imparts beneficial outcomes in cardiac repair [115, 116]. Acid-sensitive polyketal PK3 particle–encapsuled Nox2-specific siRNA has been reported to contribute to improvement in cardiac function by silencing the Nox2 gene in cardiac macrophages [129]. Leuschner et al. evaluated the therapeutic effect of an optimized lipid NP–loaded siCCR2 on cardiac repair. siCCR2-NPs accumulate in splenic phagocytic cells when administered systemically in mice [130]. Treatment with siCCR2-NPs resulted in a marked reduction in the number of inflammatory monocytes and M1 macrophages along with significant attenuation of MI progression.

Furthermore, NPs containing pioglitazone have been demonstrated to modulate monocyte/macrophage subtype [131, 132]. Pioglitazone, a peroxisome proliferator–activated receptor-*γ* (PPAR*γ*) agonist, shows a marked impact on monocyte and macrophage polarization, transferring them into the anti-inflammatory subtype [133]. Matoba et al. performed a randomized placebo-controlled study in a mouse model of plaque rupture and suggested that PLGA NPs containing pioglitazone enhance the proportion of M2 macrophages [131]. In a similar approach, pioglitazone NPs administered intravenously to an atherosclerosis ApoE^−/−^ mouse model showed a significant reduction in the number of fibrous caps by decreasing proinflammatory monocytes along with a moderate increase in anti-inflammatory phenotypes [132].

Moreover, the timely endocytosis of dead cells by macrophages can trigger the anti-inflammatory response and transform M1 macrophages into M2 macrophages. Given these findings, a theranostic nanosystem with mimicking apoptosis was established and showed a remarkable ability in resolving inflammation and promoting cardiac repair [134].

## CONCLUDING REMARKS

Macrophages play an indispensable role in the mammalian heart and respond to both the post-MI regeneration and repair by mediating inflammation and immunity. M1 and M2 macrophages are indispensable in repairing the myocardium and in retaining functional architecture. Different populations of macrophages respond to a special stage after MI. Several studies have focused on the mechanism of macrophage polarization that is related to the cardioprotective potential. With advancement in therapeutic approaches, especially the burgeoning NP-mediated drug delivery system, modulating macrophage activation to guide cardiac repair and regeneration is now a promising therapeutic strategy. These strategies are also available to other CVDs associated with macrophages, such as atherosclerosis and myocarditis.

As this review highlights, the roles of macrophages in terms of cardiac repair and regeneration are complex. To better understand the mechanisms of macrophages during the various stages of acute and chronic myocardial disease, further research is warranted. Moreover, although specially designed nanocarriers have been applied to improve the efficiency of therapeutic agents and a major breakthrough has been reached, many challenges remain, including the off-target delivery and security issues, which require additional investigation.

## Abbreviations

Arg-1: arginase 1
CCL: chemokine (C-C motif) ligand
CDCs: cardiosphere-derived cells
CSCs: cardiac stromal cells
CVD: cardiovascular disease
CXCL: C-X-C motif chemokine
DAMPs: damage-associated molecular patterns
ECM: extracellular matrix
EVs: extracellular vesicles
GM-CSF: granulocyte macrophage colony-stimulating factor
hESC-CVPCs: human embryonic stem cell–derived cardiovascular progenitor cells
HIF: hypoxia-inducible factor
IFN: interferon
IL: interleukin
IKK*β*: IκB kinase *β*
iNOS: inducible nitric oxide synthase
I/R: ischemia/reperfusion
IRAK4: interleukin-1 receptor–associated kinase 4
IRF: interferon regulatory factor
JAK: Janus kinase
KLF-4: Krüppel-like factor 4
LDL: low-density lipoprotein
LPS: lipopolysaccharide
M-CSF: macrophage colony-stimulating factor
MI: myocardial infarction
MPS: mononuclear phagocyte system
MSCs: mesenchymal stromal cells
Mrc1: mannose receptor (CD206)
MyD88: myeloid differentiation primary response gene 88
NPs: nanoparticles
PLGA: poly(lactide co-glycolic acid)
PPAR*γ*: peroxisome proliferator–activated receptor-*γ*
SOCS3: suppressor of cytokine signaling 3
STAT: signal transducers and activators of transcription
TGF-*β*: transforming growth factor
TLR4: toll-like receptor 4
TNF-*α*: tumor necrosis factor-*α*
TRAF: TNF receptor–associated factor
TRIF: TIR domain–containing adapter-inducing interferon
